# Associations of IRAK1 Gene Polymorphisms and mRNA Expression With NMOSD Risk in the Northern Chinese Han Population

**DOI:** 10.3389/fneur.2021.661791

**Published:** 2021-08-31

**Authors:** Hongjing Yan, Ruoyi Guo, Weifeng Chen, Xutao Xi, Lianchang Wang, Jianxun Ma, Bin Li

**Affiliations:** ^1^Department of Neurology, The Second Hospital of Hebei Medical University, Shijiazhuang, China; ^2^Key Laboratory of Hebei Neurology, Shijiazhuang, China; ^3^Department of Neurology, Handan First Hospital, Handan, China; ^4^Department of Neurosurgery, The Central Hospital of Handan, Handan, China

**Keywords:** IRAK1, polymorphism, NMOSD, mRNA, TLR

## Abstract

**Objectives:** Interleukin (IL)-1 receptor-associated kinase 1 (IRAK1) is a very important immunomodulatory gene for autoimmune diseases located on the X chromosome. However, there was little study about the correlation of IRAK1 functional single nucleotide polymorphisms with mRNA expression in neuromyelitis optica spectrum disorder (NMOSD) patients. In this study, we aimed to investigate the plausible association of IRAK1 polymorphism, IRAK1 mRNA expression, and NMOSD risk in the northern Chinese Han population.

**Methods:** Four loci of IRAK1 gene (rs1059702, rs7061789, rs1059703, and rs3027898) were genotyped using multiplex SNaPshot technique in 102 NMOSD patients and 213 healthy subjects. Allele, genotype, and haplotype frequencies were compared. Stratified analyses were conducted by age, sex, AQP4 status, and age of onset. IRAK1 mRNA levels in the peripheral blood mononuclear cells of 30 NMOSD patients (of active phase) and 15 healthy control subjects were detected using qPCR. The correlations between the SNP polymorphisms and mRNA expression levels of genes were tested using non-parametric tests.

**Results:** The minor allele frequencies (MAF) of these four locis were significantly lower in NMOSD cases than that of the controls. The frequencies of rs1059703G/G genotype, rs1059702A/A genotype, rs3027898 C/C genotype, and rs7061789G/G genotype were higher in the case group than that of the control group. Haplotype analysis revealed that the major haplotype “G-A-C-G” (alleles in the order of SNPs rs1059703, rs1059702, rs3027898, and rs7061789), containing the risk alleles, conferred an adverse effect on NMOSD. The level of IRAK1mRNA was markedly higher in NMOSD when compared to the healthy control groups. The IRAK1mRNA levels of female patients with the major haplotype were significantly higher compared to those with other haplotypes and to the male patients with the same genotype.

**Conclusion:** IRAK1 polymorphisms were highly correlated with NMOSD susceptibility. Its haplotype G-A-C-G (rs1059703-rs1059702-rs3027898-rs7061789) confers increasing the risk of NMOSD in female patients. The IRAK1 risk haplotype G-A-C-G upregulated IRAK1 mRNA expression in female NMOSD patients. Our study provides a novel insight into the molecular mechanism of the pathogenesis of NMOSD and reveals that IRAK1 is the potential mechanism-specific druggable target in NMOSD disease.

## Introduction

Neuromyelitis optica spectrum disorder (NMOSD) is a demyelinating autoimmune disease primarily affecting the spinal cord and optic nerve. Compared to multiple sclerosis, Neuromyelitis optica occurs more frequently in women than in men, by as much as (1, 2): 1 (3). The primary outcomes are physical and visual disabilities. Since the discovery of anti-AQP4 antibodies (AQP4-IgG) in 2004 (4), it is generally believed that NMOSD is characterized by the AQP4-IgG, which are produced by the differentiation of B cells to plasma cells (5, 6), binding to AQP4 on astrocytes (4, 7), and cause direct astrocyte injury by antibody-dependent and complement-mediated (cellular) cytotoxicity. However, the exact molecular mechanisms behind the pathogenesis of NMOSD remain unclear. Some studies have shown that toll-like receptor (TLR) signaling, which has been implicated in various neuroimmune processes, may play a relevant role in the pathogenesis of NMOSD (1, 2, 8).

Interleukin (IL)-1 receptor-associated kinase 1(IRAK1), which is a vital serine/threonine-protein kinase and the key signal regulator, plays an indispensable role in scaffolding and phosphorylation in TLR signaling pathways (9–13). Recent studies observed an abnormal expression of IRAK1 in several autoimmune diseases, including systemic lupus erythematosus (SLE) and rheumatoid arthritis (RA) (14, 15). Zhou et al. found that the levels of IRAK1 transcript in CD4+T cells increased significantly in patients with SLE and were positively associated with disease activity (14). Another work from Ji et al. indicated that inhibition of IRAK1-NF-κB signaling activity might attenuate the inflammatory activity of MRL/lpr mice and their bone marrow-derived macrophages (15). A previous *in vivo* experiment disclosed that iguratimod reduced systemic arthritis score, and decreased IRAK1, IL-6, IL-1β, TNF-α, and IL-17, but enhanced apoptosis in the synovial tissue of a CIA rat model (16). Collectively, these results suggest that IRAK1 may play a vital role in autoimmune diseases, and further study of IRAK1 is warranted.

The IRAK1 gene is located at human locus Xq28. In women, one of the two X chromosomes which are from each of the two parents, undergoes random X-inactivation in the early stages of embryonic development. Males carry and express only one ChrX, and X chromosome inactivation (XCI) in females provides dosage compensation between the sexes for X-linked genes (17, 18). Several studies have demonstrated that the IRAK1 polymorphism may be related to autoimmune diseases, including SLE, RA, and autoimmune thyroid diseases (19–23). These common loci including rs1059702, rs1059703, rs3027898, and rs7061789 were found to be in strong linkage disequilibrium (22, 24). Furthermore, these SNPs may be functional SNPs that are likely to play a significant role in modulating the phenotype in females. For example, studies have demonstrated that rs3027898 may be a functional SNP that can regulate IRAK1 mRNA expression (25, 26). Another study showed that rs1059703 may regulate skewed X chromosome inactivation which is likely to play a vital role in modulating the causes of augmented cell activation (27).

IRAK1 is a gene central to immune regulation. However, its role is less studied in NMOSD. It would be useful to explore the SNP genotypes in IRAK1 and its relation to mRNA expression analysis, and their association with NMSOD. Our study identifies that IRAK1 polymorphisms are correlated with NMOSD risk in northern Chinese Han populations and contribute to the up-regulated expression level of IRAK1 mRNA in female NMOSD patients.

## Materials and Methods

### Study Subjects

We enrolled patients diagnosed with NMOSD at the Department of Neurology in the Second Hospital of Hebei Medical University (102 patients including 86 females and 16 males, 30 acute patients including 25 females and five males) from September 2019 to November 2020. Meanwhile, 213 healthy examinees (166 females and 47 males) were enrolled as a healthy control group at the physical examination center. All subjects were from the northern Han Chinese population. It is worth noting that we only enrolled NMOSD patients who fulfilled the 2015 diagnostic criteria for NMOSD (27). Subjects used for determining IRAK1 mRNA expression were active NMOSD patients who had not used hormone or immunomodulatory agents in the previous 6 months. The following exclusion criteria were prescribed: (a) hypertension, diabetes, cerebrovascular disease, tumors, and other chronic diseases; and (b) concurrent with other autoimmune diseases, such as rheumatoid arthritis, systemic lupus erythematosus, and thyroid diseases. All participants signed an informed consent before the study began. Data collection involved extracting relevant details from the patients into a standard case report form, including age, gender, age at onset, AQP4 status, clinical manifestations and initial symptoms, medication history, and disease history. This study was approved by the Ethics Committee of The Second Hospital of Hebei Medical University.

### Isolation of DNA and Genotyping by SNaPshot Technique

We collected 5 ml of peripheral blood in EDTA anticoagulant tubes from each subject. Next, we extracted genomic DNA using the Blood Genomic DNA Extraction Kit (JieRui, Shanghai, China) according to the manufacturer's protocol, and stored it at −20°C for subsequent experiments. DNA was amplified using PCR MIX (Yisheng Biotech Co., Shanghai, China), with focus on four IRAK1 sites (rs1059703-rs1059702-rs3027898-rs7061789).The Snapshot kit (ABI, USA) was used for genotyping (28). Table 1 shows the sequences of primers.

**Table 1:** Primer information.

**Genes**	**Primer sequence**	
	**Upstream primer**	**Downstream primer**
rs1059702	CTCCTCCGAGAAGTTGTG	GCAGAAGGGAAGAGACAG
rs1059703	CTGGACACGTAGGAGTTC	TGTACGAGAGGCTAGAGAA
rs3027898	AACATGGACTACATCAGGAA	GGCTCTACTTGTGGACTT
rs7061789	AACATGGACTACATCAGGAA	TGATTTCAGAACTGGAGATG
IRAK1	AAGGAGAACGCTGACCTGGAGT	CCGTACACCAGGCAGTAGAAGC
β-actin	CACCCAGCACAATGAAGATCAA	CCAGTTTTTAAATCCTGAGTCAAGC

### qPCR for IRAK1 mRNA

Furthermore, we enrolled 30 active NMOSD patients and 15 healthy normal controls for examining IRAK1 mRNA expression. We collected a 5 ml fasting EDTA-anticoagulated venous blood sample from each subject. We then isolated peripheral blood mononuclear cells by density-gradient centrifugation using Ficoll–Hypaque (Hao Yang Biological, Tianjin, China). Next, total RNA was isolated using the Total RNApure reagent (ZhuangMeng Biological, Beijing, China) in accordance with the manufacturer's protocol. Complementary DNA (cDNA) was then synthesized from RNA using Prime Script™ RT reagent Kit with gDNA Eraser (GeneCopoeia, Guangzhou, China) according to the manufacturer's instructions. Real-time quantitative PCR (qPCR) involved the use of qPCR Mix (GeneCopoeia, Guangzhou, China) and was performed on an ABI7300 machine. PCR amplification conditions were: 95°C pre-denaturation for 10 min, 95°C for 15 s, 60°C for 5 min, and 75°C for 20 s, repeated for 40 cycles. Two compound holes were set for each hole. Relative mRNA expression was normalized to the expression of IRAK1 mRNA, and the 2-ΔΔCt method was used to calculate the relative quantification of the gene expression level (28).

### Statistics

Results are presented as number (percentage) for frequency and as mean ± standard deviation for quantitative variables. We used the unpaired Student's *t*-test to compare the differences in means between two groups, and chi-squared tests for categorical variables. Logistic regression analysis was performed to determine the association of IRAK1 SNPs with the risk factors, while one-way analysis of variance (ANOVA) with Bonferroni's *post-hoc* test was used to compare the differences in means among multiple groups. Moreover, we used Pearson's correlations to analyze bivariate correlations. The online software SHEsis (http://analysis2.bio-x.cn/myAnalysis.php) was used for linkage disequilibrium (LD), Hardy-Weinberg balance test, and haplotype (23). SNPStats (https://www.snpstats.net/start.htm) was used to construct haplotypes and analyze the interactions with related factors. The computed statistical power for this study was 0.88 (*n* = 126, P0 = 0.2, α = 0.05, OR = 2) using PASS 15.0. All other statistical analyses were performed using SPSS version 21.0 (IBM Corp., Armonk, NY, USA). *P* < 0.05 was considered to be statistically significant.

## Results

### Analysis of Clinical Data

Tables 2, 3 show the characteristics of study participants. In total, we analyzed 315 study participants for SNP, including 213 healthy controls (female 77.9%) and 102 NMOSD patients (female 84.3%). The mean age of NMOSD patients was 45.04 ± 10.86 years, while that of the control group was 44.34 ± 16.82 years. This indicates that the mean age or gender distribution was not significantly different between the case and control groups (*p* = 0.95). Mean age at onset was 43.63 ± 16.81. Results indicated that serum anti-AQP4 antibodies were positively detected in 87 (85.9%) patients, optic neuritis in 22 (23.3%), myelitis in 48.54%, and brain attacks and mixed attacks in 34 (28.16%). Furthermore, we collected peripheral blood samples from 30 active NMOSD patients (female 25 cases, male five cases) and 15 healthy controls for IRAK1 mRNA expression analysis. Their mean age was 42.5 ± 15.507 and 39.6 ± 9.553, respectively, which showed no statistical significance. We positively detected serum AQP4-IgG in 23 (76.7%) patients, optic neuritis in 7 (23.3%) patients, myelitis in 16 (53.3%) patients, brain attacks in 3 (10%) patients, and mix attacks in 4 (13.3%) patients.

**Table 2:** Demographics and clinical characteristics of participants for IRAK1 SNP.

	**NMOSD**	**HC**	***P*** **-values**
	***n*** **= 102**	***n*** **= 213**	
Sex, no. (%) of females	84.3%	77.9%	0.185
Age, y (mean ± SD)	45.04 ± 10.86	44.93 ± 16.82	0.95
Age at onset, y (mean ± SD)	43.63 ± 16.18	NA	NA
AQP4-IgG+, no. (%) of patients	85.29%	NA	NA
**Onset symptoms, no. (%) of patients**			
Optic neuritis	23.30%	NA	NA
Acute myelitis	48.54%	NA	NA
Brain attacks	10.67%	NA	NA
Mix attacks	17.49%	NA	NA

*NMOSD, Neuromyelitis optica spectrum disorder; HC, Healthy control group; SD, standard deviation; NA, not assessable. Brain attacks, including the brainstem and brain attacks*.

**Table 3:** Demographics and clinical characteristics of participants for IRAK1 mRNA.

	**NMOSD**	**HC**	***P*** **-values**
	***n*** **= 30**	***n*** **= 15**	
Sex, no. (%) of females	83.3%	66.6%	0.205
Age, y (mean ± SD)	42.5 ± 15.507	39.6 ± 9.553	0.444
AQP4-IgG+, no. (%) of patients	51.1%	NA	NA
**Onset symptoms, no. (%) of patients**			
Optic neuritis	23.30%	NA	NA
Acute myelitis	53.3%	NA	NA
Brain attacks	6.7%	NA	NA
Mix attacks	8.9%	NA	NA

*NMOSD, neuromyelitis optica spectrum disorders; HC, healthy controls; SD stands for standard deviation; NA stands for data not available. Brain involvement includes the involvement of the brain stem, cerebrum, and cerebellum*.

### Genotyping and HWE

In this study, we detected four loci (rs1059702, rs7061789, rs1059703, and rs3027898) on the IRAK1 gene. Given that IRAK1 was located on the X chromosome, we tested all the SNPs for Hardy–Weinberg equilibrium (HWE) only in female participants. Genotypic distributions for all loci were in Hardy–Weinberg equilibrium (*p* > 0.05 for all), suggesting that the selected sample population was representative (Table 4).

**Table 4:** Selected SNPs of IRAK1 in this study.

**SNPs**	**Location**	**Group (*n*)**	**Genotype (***n***)**			***P***_**HWE**_	**MAF**	**Nucleotide**	**Functional region**
rs1059702	X:154018741 (GRCh38.p12)	Case (*n* = 86)	AA 66	AG 18	GG 2	*P* = 0.56	0.17	A>G	Exon6
		Control (*n* = 166)	102	51	13	*P* = 0.07			
rs1059703	X:154013378 (GRCh38.p12)	Case (*n* = 86)	AA 2	AG 18	GG 66	*P* = 0.15	0.25	G>A	Exon12
		Control (*n* = 166)	12	52	102	*P* = 0.56			
rs3027898	X:154010439 (GRCh38.p12)	Case (*n* = 86)	AA 3	AC 16	CC 67	*P* = 0.12	0.25	C>A	Downstream
		Control (*n* = 166)	10	50	106	*P* = 0.22			
rs7061789	X:154015024 (GRCh38.p12)	Case (*n* = 86)	AA 2	AG 17	GG 67	*P* = 0.47	0.24	G>A	Intron
		Control (*n* = 166)	10	48	108	*P* = 0.14			

*SNPs, single-nucleotide polymorphisms; P_HWE_, p-value for Hardy-Weinberg equilibrium; MAF, minor allele frequency*.

### Correlation Analysis Between rs1059703, rs7061789, rs3027898, or rs1059702 and the NMOSD Risk in Females Under Different Models

Because of the small sample size of males (with only 16 males), we did not compare data statistically for SNPs on males. Table 5 shows the genotype and allele frequency of the tested four SNPs in the NMOSD case group and the control group. Take rs1059702 A>G for example, the allele A was the dominant allele due to higher frequency (76.8%) compared to the allele G (12.8%). The minor allele frequency (MAF) allele G was significantly lower in cases than in controls (OR = 0.486; 95% CI, 0.290–0.813; *p* = 0.006). In the inheritance dominant model, patients with the AG+GG genotype had a statistically significantly higher risk of NMOSD than healthy controls with the AA genotype after adjusting for age (OR = 0.48, 95% CI = 0.27–0.87, *p* = 0.013). Similarly, rs7061789, rs1059703, and rs3027898 also showed significantly different alleles and genotype distributions among female patients with NMOSD and healthy controls.

**Table 5:** Association analysis between SNPs and the female NMOSD risk under different models.

**Model**	**Genotype**	**Control**	**Case**	**OR (95% CI)**	***P*** **-value**
**rs1059703**	G	256 (77.1%)	150 (87.2%)	0.49 (0.29–0.82)	0.007
Allele	A	76 (22.9%)	22 (12.8%)	1	
Codominant	G/G	102 (61.5%)	66 (76.7%)	1	0.029
	A/G	52 (31.3%)	18 (20.9%)	0.53 (0.29–0.99)	
	A/A	12 (7.2%)	2 (2.3%)	0.26 (0.06–1.19)	
Dominant	G/G	102 (61.5%)	66 (76.7%)	1	0.013
	A/G-A/A	64 (38.5%)	20 (23.3%)	0.48 (0.27–0.87)	
Recessive	G/G-A/G	154 (92.8%)	84 (97.7%)	1	0.084
	A/A	12 (7.2%)	2 (2.3%)	0.31 (0.07–1.40)	
Overdominant	G/G-A/A	114 (68.7%)	68 (79.1%)	1	0.076
	A/G	52 (31.3%)	18 (20.9%)	(0.31–1.07)	
**rs1059702**	A	255 (76.8%)	150 (87.2%)	0.48 (0.29–0.81)	0.006
Allele	G	22 (23.2%)	77 (12.8%)	1	
Codominant	A/A	102 (61.5%)	66 (76.7%)	1	0.025
	A/G	51 (30.7%)	18 (20.9%)	0.55 (0.29–1.01)	
	G/G	13 (7.8%)	2 (2.3%)	0.24 (0.05–1.09)	
Dominant	A/A	102 (61.5%)	66 (76.7%)	1	0.013
	A/G-G/G	64 (38.5%)	20 (23.3%)	0.48 (0.27–0.87)	
Recessive	A/A-A/G	153 (92.2%)	84 (97.7%)	1	0.059
	G/G	13 (7.8%)	2 (2.3%)	0.28 (0.06–1.27)	
Overdominant	A/A-G/G	115 (69.3%)	68 (79.1%)	1	0.094
	A/G	51 (30.7%)	18 (20.9%)	0.60 (0.32–1.10)	
**rs3027898**	C	262 (78.9%)	150 (87.2%)	0.54 (0.32–0.92)	0.024
Allele	A	70 (21.1%)	22 (12.8%)	1	
Codominant	C/C	106 (63.9%)	67 (77.9%)	1	0.068
	A/C	50 (30.1%)	16 (18.6%)	0.51 (0.27–0.96)	
	A/A	10 (6%)	3 (3.5%)	0.47 (0.13–1.79)	
Dominant	C/C	106 (63.9%)	67 (77.9%)	1	0.02
	A/C-A/A	60 (36.1%)	19 (22.1%)	0.50 (0.27–0.91)	
Recessive	C/C-A/C	156 (94%)	83 (96.5%)	1	0.37
	A/A	10 (6%)	3 (3.5%)	0.56 (0.15–2.11)	
Overdominant	C/C-A/A	116 (69.9%)	70 (81.4%)	1	0.044
	A/C	50 (30.1%)	16 (18.6%)	0.53 (0.28–1.00)	
**rs7061789**	G	264 (79.5%)	149 (87.8%)	0.54 (0.31–0.91)	0.022
Allele	A	151(20.5%)	21(12.2%)	1	
Codominant	G/G	108 (65.1%)	67 (77.9%)	1	0.079
	A/G	48 (28.9%)	17 (19.8%)	0.57 (0.30–1.07)	
	A/A	10 (6%)	2 (2.3%)	0.32 (0.07–1.52)	
Dominant	G/G	108 (65.1%)	67 (77.9%)	1	0.033
	A/G-A/A	58 (34.9%)	19 (22.1%)	0.53 (0.29–0.96)	
Recessive	G/G-A/G	156 (94%)	84 (97.7%)	1	0.17
	A/A	10 (6%)	2 (2.3%)	0.37 (0.08–1.73)	
Overdominant	G/G-A/A	118 (71.1%)	69 (80.2%)	1	0.11
	A/G	48 (28.9%)	17 (19.8%)	0.61 (0.32–1.13)	

### Linkage Disequilibrium and Haplotype Analysis

**Figure 1:**
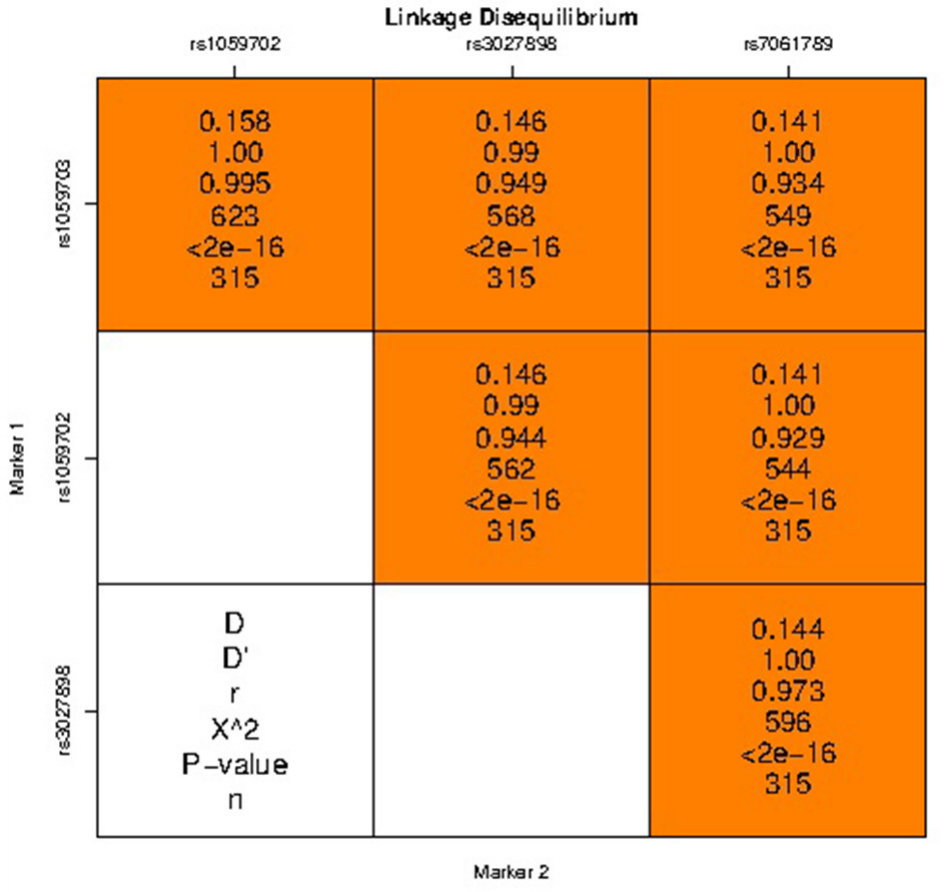
Linkage disequilibrium diagram of four SNP loci in the IRAK1 gene.

The haplotype analysis showed that rs1059702, rs7061789, rs1059703, and rs3027898 were in high linkage disequilibrium (Figure 1). Haplotype analysis identified two common haplotypes with a frequency higher than 0.03: haplotype1 = GAGC with a frequency of 87.75% in the NMOSD case group and 76.29% in the healthy control group, and haplotype2 = AGAA with a frequency of 10.29% in the NMOSD case group and 21.13% in the healthy control group. There was a statistically significant difference in haplotype distribution between NMOSD cases and healthy controls (*p* = 0.0061) (Table 6).

**Table 6:** IRAK1 haplotype association with NMOSD.

	**rs1059703**	**rs1059702**	**rs3027898**	**rs7061789**	**Control**	**Case**	**OR (95% CI)**	***P*** **-value**
**1**	**G**	**A**	**C**	**G**	0.7629	0.8775	1	—
**2**	**A**	**G**	**A**	**A**	0.2113	0.1029	0.50 (0.31–0.81)	0.0049
3	A	G	C	G	0.0188	0.0049	0.25 (0.03–2.05)	0.2
Rare	^*^	^*^	^*^	^*^	^*^	^*^	2.08 (0.48–8.99)	0.33

*Global haplotype association p-value: 0.0061, (n = 313, adjusted by sex + age)*.

* *represent data is not available. The bold font refers to the statistical significance of result*.

### Stratification Analysis Based on Clinical Features of Female NMOSD Patients

We conducted statistical analysis to evaluate the significance of IRAK1 for SNPs to the listed clinical characteristics (AQP4 status, onset age, onset symptoms). However, no statistically significant difference was observed in genotype and allele frequencies after stratification by clinical characteristics (Table 7).

**Table 7:** Stratified analysis of IRAK1-four SNPs in different clinical features of patients with female NMOSD.

**Clinical character**	**Genotype**	**Phenotype**	**Phenotype**	**OR (95% CI)**	***P*** **-value**
AQP4 status		AQP4IgG-	AQP4IgG+		
rs1059703	G/G	7 (70%)	71 (79.8%)	1	0.49
	A/G-A/A	3 (30%)	18 (20.2%)	0.59 (0.14–2.52)	
rs1059702	A/A	7 (70%)	71 (79.8%)	1	0.49
	A/G-G/G	3 (30%)	18 (20.2%)	0.59 (0.14–2.52)	
rs3027898	C/C	7 (70%)	72 (80.9%)	1	0.44
	A/C-A/A	3 (30%)	17 (19.1%)	0.55 (0.13–2.35)	
rs7061789	G/G	7 (70%)	73 (82%)	1	0.38
	A/G-A/A	3 (30%)	16 (18%)	0.51 (0.12–2.19)	
age of onset		≤ 43	>43		
rs1059703	G/G	43 (82.7%)	35 (74.5%)	1	0.32
	A/G-A/A	9 (17.3%)	12 (25.5%)	1.64 (0.62–4.33)	
rs1059702	A/A	43 (82.7%)	35 (74.5%)	1	0.32
	A/G-G/G	9 (17.3%)	12 (25.5%)	1.64 (0.62–4.33)	
rs3027898	C/C	43 (82.7%)	36 (76.6%)	1	0.45
	A/C-A/A	9 (17.3%)	11 (23.4%)	1.46 (0.54–3.91)	
rs7061789	G/G	43 (82.7%)	37 (78.7%)	1	0.62
	A/G-A/A	9 (17.3%)	10 (21.3%)	1.29 (0.47–3.52)	
**Onset symptoms**					
rs1059703	G/G	78	1.47 (0.13)	0	0.61
	A/G-A/A	21	1.62 (0.25)	0.14 (−0.41 - 0.70)	
rs1059702	A/A	78	1.47 (0.13)	0	0.61
	A/G-G/G	21	1.62 (0.25)	0.14 (−0.41 - 0.70)	
rs3027898	C/C	79	1.49 (0.13)	0	0.85
	A/C-A/A	20	1.55 (0.26)	0.06 (−0.51 - 0.62)	
rs7061789	G/G	80	1.49 (0.13)	0	0.76
	A/G-A/A	19	1.58 (0.27)	0.09 (−0.48 - 0.67)	

*AQP4IgG+, AQP4IgG positive; AQP4IgG-, AQP4IgG negative; Age at onset Mean ± SD, years = 43.63 ± 13.32 years. For stratified according to Onset symptoms set 0= optic neuritis, 1= myelitis, 2 = Mix attacks*.

### Analysis of IRAK1 mRNA Expression

**Figure 2:**
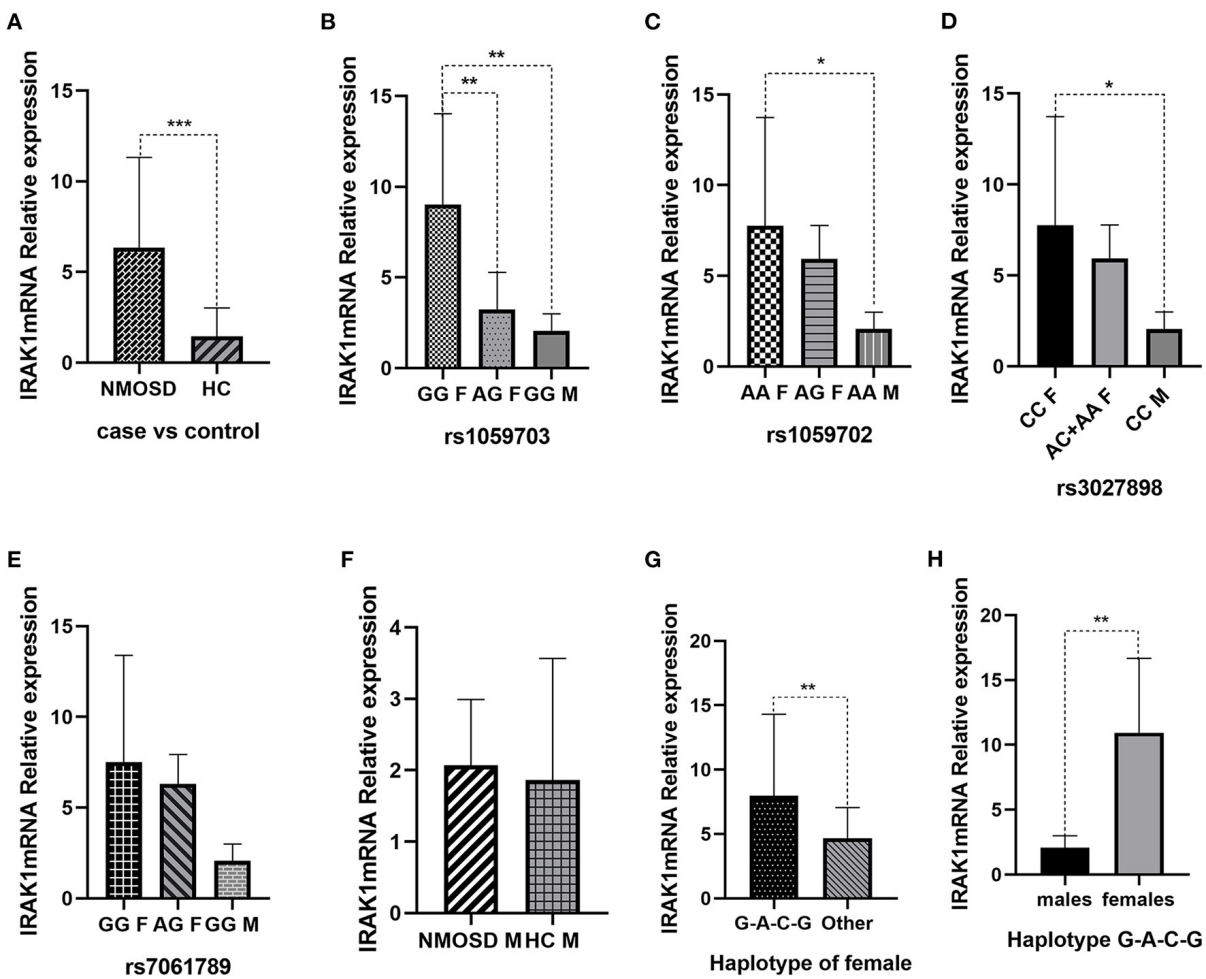
**(A)** Expression of IRAK1 mRNA level in NMOSD case group and healthy control group. **(B)** Expression of IRAK1 mRNA level in NMOSD patients with different rs1059703-genotypes. **(C)** Expression of IRAK1 mRNA level in NMOSD patients with different rs1059702-genotypes. **(D)** Expression of IRAK1 mRNA level in NMOSD patients with different rs3027898-genotypes. **(E)** Expression of IRAK1 mRNA level in NMOSD patients with different rs7061789-genotypes. **(F)** Expression of IRAK1 mRNA level in male NMOSD patients and male healthy control group. **(G)** Expression of IRAK1 mRNA level in female NMOSD patients with different haplotypes. **(H)** Expression of IRAK1 mRNA level in male and female NMOSD patients with the risk haplotype. ^*^P < 0.05, ^**^P < 0.01, ^***^P < 0.001.

The relative expression level of IRAK1 mRNA in the NMOSD case group (Figure 2), was statistically significantly higher than in the healthy control group (*p* = 0.003). The level of IRAK1 mRNA expression was significantly higher in the patents with rs1059703-GG genotype than that with AG genotype (*p* = 0.005, Figure 2).The level of IRAK1 mRNA expression was also significantly higher in female NMOSD patients carrying the risk haplotype GACG (rs1059703-rs1059702-rs3027898-rs7061789) than in those carrying the other haplotypes (*p* = 0.004). In all genotypes, there were increased IRAK1 mRNA expression levels in female NMOSD cases, as compared to males (*p* < 0.05, *p* < 0.01).However, no significant differences were observed between male individuals with the GG genotype in the NMOSD case group and in the healthy control group (*p* > 0.05).

## Discussion

The well-established fact that X-linked IRAK1 polymorphisms host susceptibility to autoimmune diseases prompts us to investigate the relevance of IRAK1 gene polymorphism with the risk of NMOSD. In our study, genotype polymorphisms of four common sites in the IRAK1 gene of the northern Chinese Han population were evaluated. The results indicate that both allele and genotype frequencies of the four sites had significant differences between case and control groups. Also, strong linkage disequilibrium is observed among four polymorphisms. For example, the minor allele frequency (12.8%) in the SNP of rs1059702A>G is lower in NMOSD cases than in controls. Furthermore, the AA genotype of rs1059702 may be a potential risk factor of NMOSD. The lower frequency of the minor allele suggests a protective effect of the minor allele against NMOSD, while the major allele was the risk allele. Subsequent haplotype analysis shows that the haplotypes GACG (rs1059703-rs1059702-rs3027898-rs7061789) confers increasing the risk of NMOSD.

Recently, Zhou et al. also found a similar risk association between IRAK1 polymorphisms (rs1059703, rs3027898) and NMOSD (29). The allelic frequencies of rs1059703 and rs3027898 and the linkage disequilibrium relationship between the two SNPs in the control group obtained in our study are quite similar to those described by Zhou et al. (29). Due to a relatively small volume of the case group, the MAF of rs1059703 and rs3027898 was higher in our study compared to those described by Zhou et al., but we achieved a statistical power of 88%. The subjects enrolled in our study were from the northern Han population and those in the study of Zhou et al. were from the Han population from southwest China. Our results agree with those from Zhou. Taken together, the two studies evaluated the association of two common polymorphisms (rs1059703 and rs3027898) in the IRAK1 gene with the risk of NMOSD for Chinese.

Multiple IRAK1 polymorphisms have also been reported to be associated with the susceptibility of several other autoimmune disorders. For example, in a rheumatoid arthritis susceptibility study, the MAF of SNP rs3027898 in the rheumatoid arthritis patient group was significantly higher than in healthy controls and associated with increasing susceptibility to this disease (30). Similarly, the same minor allele has been associated with increasing susceptibility to MS, SLE (23), autoimmune thyroid diseases (29), and rheumatoid arthritis (31). The two polymorphisms, rs1059703 and rs1059702, which are missense mutations and in strong linkage disequilibrium, are associated with autoimmune disease. A previous study showed that rs1059703 T major alleles were increased in RA patients, suggesting T was a risk factor for RA in both Tunisian and French women and the rs1059702 C major allele was associated with RA in French women (20). These studies together with the present results indicate that IRAK1 may be a new gene maker of NMOSD. This may be tied to their underlying roles in inflammation disease pathogenesis. IRAK1 possibly plays a key role in the signaling pathways of Toll-like receptors/IL-1R, respectively, activates NF-κB signaling pathways and the release of various inflammatory factors such as IL6, IL-1β, and TNFα, which are vital in innate immunity for the molecular and cellular mechanisms of NMOSD. The exact mechanism awaits further investigation.

The tested gene polymorphism loci have been found in the IRAK-1 gene coding and non-coding area, and in one intron area. Although there are few studies of IRAK1 polymorphisms and the association between IRAK1 gene expression in NMOSD, there were several studies showing that rs3027898 was associated with significant downregulation of IRAK1 mRNA levels (25), and the risk genotype of rs1059702 appeared to act to decrease the mRNA levels of MECP2 (included in IRAK1) in SLE patients (26). Rs3027898 was located on 3′UTR area of IRAK1. Single-nucleotide polymorphisms located in the 3′UTR may influence gene expression by varying the interaction between the mRNA and their regulation genes. Thus, to test whether these SNPs associate with mRNA-expression levels, we examined the mRNA expression of IRAK1 and linked SNP haplotypes with the IRAK1 mRNA expression level. The result showed IRAK1 mRNA expression level was affected by rs1059703 genotype, haplotype, and sex. The IRAK1 mRNA expression level was significantly higher in female NMOSD patients carrying the risk haplotype than in those carrying the other haplotypes. This could be due to the effect of the risk haplotype on the interaction between the mRNA and their regulation genes. IRAK1 mRNA expression level was not upregulated in the risk haplotype carrier of males, indicating that IRAK1 polymorphism may regulate mRNA expression by a variety of mechanisms including the skewing of X chromosome inactivation. Certain Xic mutations or polymorphisms can result in X chromosome inactivation skewing, which may result in misexpression between the sexes in diseases. A recent study showed that altered X-chromosome inactivation in T cells may be associated with sex-biased autoimmune diseases (32). In mammals, X chromosome inactivation (XCI) seems to be a mechanism to compensate for the dosage difference of X-linked genes between females (XX) and males (XY). It has also been suggested that X-linked genetic polymorphisms together with the skewing of XCI may be the reason for sex-related differences in the phenotype in the female (33). A recent study on trauma showed that the variant IRAK1 haplotype (a risk allele carrier) caused augmented cell activation which may impact cell trafficking during the trauma course (27). Previous animal experiments revealed functional variability in cellular mosaicism for IRAK1 expression and natural X-linked polymorphisms during sepsis. The exact mechanisms of IRAK1 DNA polymorphisms regulating IRAK1 mRNA expression are still not known. Further study focusing on IRAK1 SNP function is highly desirable in the future.

The actual number of NMOSD cases in our sample is somewhat on the low side; in particular, male patients are difficult to recruit because of the low disease incidence in the population. Nevertheless, there is no published report on the association between the IRAK1 haplotype GACG (rs1059703-rs1059702-rs3027898-rs7061789) polymorphisms and the mRNA expression of this gene, and our attempt is the first study in this topic. Furthermore, there is currently no evidence that IRAK1 polymorphisms may regulate the inactivation of genes on the X chromosome. We still need the validation cohort study to further confirm our results.

In conclusion, IRAK1 polymorphisms were highly correlated with NMOSD susceptibility. Its haplotype GACG (rs1059703-rs1059702-rs3027898-rs7061789) confers increasing the risk of NMOSD in females. The IRAK1 risk haplotype GACG might upregulate IRAK1 mRNA expression in female NMOSD patients. Our study provides a novel insight into the molecular mechanism of the pathogenesis of NMOSD and reveals that IRAK1 is the potential mechanism-specific druggable target in NMOSD disease.

## Data Availability Statement

The data presented in the study are deposited in the https://www.ebi.ac.uk/eva repository, accession number Project: PRJEB46209, Analyses: ERZ2822693.

## Ethics Statement

The studies involving human participants were reviewed and approved by the Clinical Research Ethical Committee of the Second Hospital of Hebei Medical University. The patients/participants provided their written informed consent to participate in this study.

## Author Contributions

BL designed the research, reviewed, revised, and edited the article. BL and HY conducted and coordinated the research. WC and RG performed the analysis and modeling of the data. HY drafted the article. XX and LW contributed to the Materials and Methods section and the figures. All authors contributed to the research and writing of the article.

## Conflict of Interest

The authors declare that the research was conducted in the absence of any commercial or financial relationships that could be construed as a potential conflict of interest.

## Publisher's Note

All claims expressed in this article are solely those of the authors and do not necessarily represent those of their affiliated organizations, or those of the publisher, the editors and the reviewers. Any product that may be evaluated in this article, or claim that may be made by its manufacturer, is not guaranteed or endorsed by the publisher.
